# Wafer Defect Recognition for Industrial Inspection: FCS-VMamba Model and Experimental Validation

**DOI:** 10.3390/jimaging12040142

**Published:** 2026-03-24

**Authors:** Yijia Zhang, Ziyi Ma, Tongji Cui, Tiejun Zhao, Qi Wang, Jianhua Wang

**Affiliations:** 1 School of Materials Science and Engineering, Hebei University of Technology, Tianjin 300401, China; oneplusz66@163.com; 2 School of Artificial Intelligence and Data Science, Hebei University of Technology, Tianjin 300401, China; 3 Arizona College of Technology, Hebei University of Technology, Tianjin 300401, China; 225674@stu.hebut.edu.cn; 4 Chinacoal Beijing Coal Mining Machinery Co., Ltd., Beijing 102400, China; zhaotiejun@chinacoal.com; 5 Xuzhou XCMG Excavating Machinery Co., Ltd., Xuzhou 221131, China; wangqi2103@163.com; 6 School of Computer Science, Inner Mongolia University, Hohhot City 010031, China; jhwang@imu.edu.cn

**Keywords:** wafer defect recognition, visual state space model, frequency attention, cross-layer cross-attention, saliency feature suppression, parameter-efficient network

## Abstract

In industrial imaging scenarios, semiconductor wafer defect classification is crucial for chip manufacturing yield and reliability. However, numerous challenges persist, including weak imaging responses and detail loss during downsampling, complex backgrounds that interfere with feature extraction, and the trade-off between performance and efficiency on edge devices. Traditional CNNs and ViTs exhibit limitations in modeling long-range dependencies and managing edge deployment costs. To address these issues, we leverage the VMamba architecture, a Visual State Space Model (SSM) that achieves global contextual modeling with linear computational complexity. Based on the VMamba architecture, we propose FCS-VMamba, a domain-adapted model that integrates three core modules, namely Frequency Attention (FA), Cross-Layer Cross-Attention (CLCA), and Saliency Feature Suppression (SFS). The experimental results show that FCS-VMamba achieved 86.06% macro-precision and 87.91% Top-1 accuracy with only 1.2 M parameters. These results demonstrate that FCS-VMamba provides a practical and parameter-efficient baseline for industrial wafer defect recognition.

## 1. Introduction

With the accelerated advancement of industrialization, semiconductors, as the core pillar of the modern electronic information industry, have deeply penetrated critical sectors vital to the national economy and public welfare [1,2,3]. These encompass artificial intelligence, 5G communications, autonomous driving, high-end manufacturing, and aerospace industries. In recent years, global competition surrounding advanced process technologies has intensified. Moore’s law continues to evolve toward the 3 nm, 2 nm, and even GAA transistor eras, with wafer manufacturing complexity growing exponentially [4]. A single wafer undergoes over 1000 precision processes, encompassing hundreds of critical steps including silicon ingot growth, lithography, etching, ion implantation, chemical mechanical polishing (CMP), and metallization [5]. Within this highly complex manufacturing process, even minor environmental disturbances or process parameter deviations can trigger various surface defects such as particle contamination, scratches, defects, residues, and pattern distortion [6]. These defects are extremely minute, typically ranging in size from micrometers to submicrometers, yet they can cause circuit short circuits, open circuits, leakage, or degraded electrical performance [7]. During later manufacturing stages, the four-probe method is commonly used to perform electrical testing on individual chips on wafers to determine the presence of functional defects [8]. Test results are visualized as wafer maps, with each chip color-coded according to its test status. Subsequently, specialized process engineers name and classify defects based on their spatial distribution patterns on the wafer map [9,10]. Accurate wafer pattern classification is crucial for root cause analysis, process optimization, and production line debugging [11].

Traditional inspection methods primarily rely on manual visual assessment or rule-based optical detection technologies [12]. The former suffers from subjective judgment variations and visual fatigue, struggling to meet the consistency and repeatability demands of mass production; the latter, lacking adaptive capabilities, generally exhibits insufficient recognition accuracy and a weak generalization ability when confronted with diverse defect morphologies and complex background textures [13]. With the advancement of intelligent manufacturing, automated visual inspection has emerged as a mainstream trend, imposing heightened demands on the intelligence, real-time responsiveness, and robustness of inspection systems. Deep learning has achieved significant progress in image recognition. Models such as convolutional neural networks (CNNs) can automatically extract multi-level spatial features, thereby enhancing detection accuracy to some extent [14]. However, constrained by the local receptive field characteristics of convolutional operations, CNNs possess inherent limitations in modeling long-range dependencies, making it difficult to effectively capture cross-regional structural defect patterns [15].

To address these issues, researchers introduced the visual transformer architecture based on self-attention mechanisms. Leveraging its global context modeling capability, the transformer demonstrates distinct advantages in feature representation completeness. Nevertheless, the computational complexity of standard self-attention scales quadratically with the input sequence length, incurring substantial computational overhead and memory consumption [16]. Recently, State Space Models (SSMs) have demonstrated potential to surpass transformers in computer vision tasks. The Mamba model achieves effective modeling of long-range dependencies through a selective state mechanism while maintaining linear computational complexity, positioning it as a potential alternative for efficient inference without compromising performance [17].

In practical industrial settings, semiconductor wafer defect detection technology still faces several core challenges:

- Insufficient application of novel frameworks: Research on architectures based on Mamba models within the field of semiconductor wafer defect detection remains limited.
- Challenges in detecting minute features: Certain wafer defects, such as minute particles and fine scratches, pose difficulties for mainstream architectures, often leading to weak feature responses and information loss after multiple downsampling operations.
- Complex backgrounds and interfering factors: Wafer surface textures combined with fluctuating lighting conditions complicate defect extraction and judgment.
- Trade-off between model performance and efficiency: Complex deep learning models improve feature learning but incur significant computational resource consumption and slower inference speeds, failing to meet real-time requirements.

These challenges indicate that existing methods struggle to simultaneously satisfy accuracy, robustness, and efficiency requirements in real industrial environments. In particular, there remains a clear need for lightweight architectures that can preserve fine-grained defect features, model long-range dependencies, and maintain high inference efficiency under complex background conditions. Our contributions are summarized as follows:

- We propose the parameter-efficient and high-efficiency detection network FCS-VMamba based on VMamba. This achieves enhanced adaptability for industrial defect detection in visual domains by integrating three innovative modules.
- A novel FSSLayer module is constructed, introducing a Frequency Attention (FA) module, transforming spatial domain features into the frequency domain for attention re-weighting, and enhancing sensitivity to texture details and contour edges.
- Cross-layer cross-attention (CLCA) addresses the semantic gap between deep and shallow features by establishing cross-level feature interaction.
- The Saliency Feature Suppression (SFS) mechanism actively suppresses highly activated regions to compel the model to focus on secondary features, improving robustness against occluded samples and adversarial attacks.

We conducted experiments using a dual-level evaluation strategy: a balanced subset of 902 images for ablation studies and an industrial-scale benchmark with over 10,000 samples to simulate the extreme class imbalance of real production lines. Comparative results against EdgeNeXt-Small and RepViT indicate that FCS-VMamba achieves an optimal balance among the model size, inference speed, and classification accuracy. This provides a competitive and memory-efficient baseline for the application of Mamba-based architectures in semiconductor wafer defect classification.

## 2. Related Work

### 2.1. Wafer Defect Detection and Classification

Semiconductor wafer defect detection is a critical task in industrial manufacturing. Traditional methods primarily rely on manual inspection or rule-based optical detection, which suffer from subjectivity, visual fatigue, and insufficient generalization capabilities [12]. Automated visual inspection using deep learning has emerged as a mainstream approach, improving detection accuracy and efficiency [18].

Piao et al. [19] proposed a wafer image failure mode recognition method combining the Radon transform with ensemble decision trees, validating effectiveness on the WM-811K dataset. Nakazawa and Kulkarni [20] designed an anomaly detection framework based on deep convolutional encoders and decoders, trained with synthetic defect data to identify unknown defects. Maksim et al. [21] employed CNNs integrating sparse real and synthetic data for small-sample scenarios, achieving approximately 87.8% accuracy under data scarcity. Cheon et al. [8] proposed a CNN for wafer surface defect classification, enabling unknown defect detection, with superior performance for relevant tasks. Wang et al. [22] proposed deformable convolutional networks (DC-Nets) that adaptively sample mixed defect regions, significantly improving mixed defect recognition. Saqlain et al. [23] combined CNNs with data augmentation and regularization to mitigate class imbalance, achieving an average accuracy of 96.2% across nine-class classification tasks. Saqlain et al. [23] proposed a CNN-WDI model with data augmentation, batch normalization, and spatial dropout to address class imbalances. Ji and Lee [24] used GAN-based sample augmentation, raising CNNs’ classification accuracy on WM-811K from 97.0% to 98.3%. Kim et al. [25] integrated spatial autocorrelation analysis into a generalized uncertainty decision tree model, demonstrating superior performance on real DRAM data. Shon et al. [12] introduced an unsupervised pretraining method based on CVAE with automatic data augmentation, enhancing robustness under imbalanced datasets. Doss et al. [26] proposed a ShuffleNet-v2-CNN transfer learning model, classifying Si wafer defects from the WM-811K dataset via preprocessing, augmentation, feature extraction, and classification, achieving 96.93% overall accuracy.

### 2.2. Transformer and State Space Model-Based Architectures

While CNNs excel at local feature extraction, they cannot handle long-range dependencies [15]. Visual transformer architectures have been introduced to address this limitation, providing global context modeling and improved feature representation [16]. Wei and Wang [27] proposed the Multi-Scale Fusion Transformer (MSF-Trans). This model employs CNNs to capture local features while integrating transformers to model global dependencies, achieving significant improvements on datasets with 38 defect categories. Fan et al. [28] proposed a ViT-based data augmentation framework to address class imbalance, enhancing CNN performance for wafer map defect classification and semiconductor supply chain resilience. Xu et al. [29] proposed density-guided dual-stream ViT (DG-ViT), integrating the SGGT and DAGCN with adaptive density masking and achieving 94.81% (WM-811K) and 98.54% (Mixed-WM38) accuracy for wafer defect detection. Yuan et al. [30] proposed a position-aware self-supervised framework with RingDistanceConv (RDConv), achieving 96.41% accuracy on WM-811K (eight defect classes). Gu et al. [31] proposed WamGLM (a multimodal LLM) with two-stage fine-tuning (PSCL +WaferMapVMQA Dataset), enabling multi-turn dialogue-based wafer map defect information queries. However, standard self-attention mechanisms incur quadratic computational complexity, limiting practical deployment in resource-constrained industrial environments.

State space models (SSMs), such as the Mamba model, achieve effective long-range dependency modeling with linear computational complexity, offering a promising alternative for efficient inference [17]. Wang et al. [32] proposed MemoryMamba (a memory-augmented SSM), outperforming existing methods across four industrial datasets for defect recognition. Du et al. [33] proposed a climbing robot-based ring vision system and PD-Mamba (Vision Mamba), achieving a 77.97% mIoU and 88.81% mP for bridge pier defect detection with linear complexity. Li et al. [34] proposed a stripe-aware CNN-Mamba hybrid network (SA-CMamba) with SAEM, achieving a 90.97%, 79.24%, and 62.33% mIoU on NEU-Seg, DAGM, and CRACK500 for pixel-level surface defect detection. Yu et al. [30] proposed hierarchical Vision Mamba with adaptive multi-scale fusion (HiAM-Mamba), achieving 100% (NEU-CLS) and 99.26% (X-SDD) accuracy for steel surface defect classification. Zhang et al. [35] proposed DPFNet, a dual-path fusion network combining VisionMamba’s state space modeling with separable convolutions, achieving 98.84% accuracy on the MixedWM38 dataset.

### 2.3. Automated Defect Classification Systems

Automated defect classification (ADC) systems have been widely adopted in semiconductor manufacturing. These systems detect potential defect regions, record their coordinates, and direct high-precision SEM imaging to generate local high-resolution image blocks [8]. Despite high automation, classification accuracy remains suboptimal, often requiring subsequent manual review by experienced technicians. Recent studies have leveraged high-resolution imaging, advanced image processing, and deep learning to improve precise extraction, localization, fine-grained classification, and cause inference for wafer surface defects [36].

Early research in wafer defect detection and classification includes Liu et al. [37], who proposed a knowledge-based intelligent WBM defect diagnosis system integrating spatial statistics testing, CNN, ART neural networks and MI, validated with 12 real WBM patterns and implemented in a Taiwanese semiconductor company. Subsequently, Kung et al. [38] developed a machine learning-based framework combining text mining, image processing, deep learning, and decision rules for wafer pattern classification and auto disposition, reducing the hold time for specific hold codes by 70%. Later, Kim et al. [39] proposed Bin2Vec (a neural network-based bin coloring method) and a CNN-based WBM classification model, which outperformed machine learning benchmarks on 27,701 real WBMs with improved visualization effects. Dey et al. [40] proposed an ensemble deep learning framework integrating RetinaNet with multiple backbones, using preference-weighted aggregation and unsupervised denoising to improve complex defect detection. Yoon and Kang [41] developed a semi-automated classification system combining CNN predictions with expert intervention, achieving over 99% accuracy and 93% coverage. Yang and Sun [42] explored a classical quantum hybrid deep learning paradigm, demonstrating wafer image classification and hotspot detection on approximate quantum processors for the first time. More recently, Dey et al. [40] put forward an ensemble deep learning model integrating RetinaNet backbones and preference-based fusion combined with unsupervised denoising to enhance the mAP for defect classification, detection, and localization in SEM images, while Neumann et al. [43] proposed an ML-based automated application for defect detection and classification from the ZEISS MultiSEM^®^ (Carl Zeiss, Oberkochen, Germany) images of imec iN5 node semiconductor wafers.

## 3. Methodology

### 3.1. Overall Workflow

**Figure 1:**
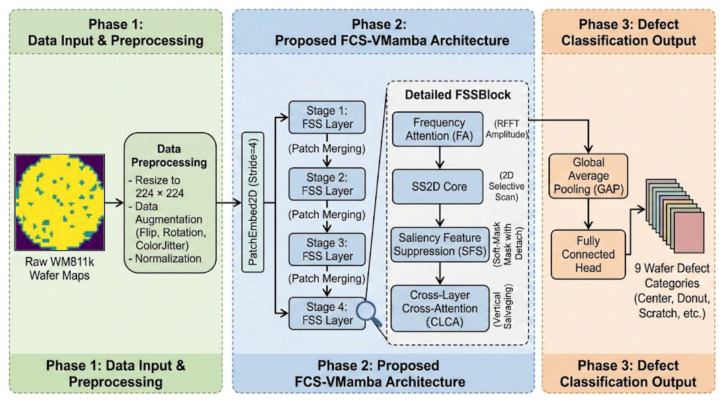
Overall workflow of the proposed FCS-VMamba architecture.

To address the complexity of semiconductor wafer defect detection, we propose the FCS-VMamba architecture. To provide a clear and comprehensive understanding, Figure 1 illustrates the overall workflow of our proposed method, which is logically divided into three primary phases: data input and preprocessing, FCS-VMamba feature extraction, and defect classification output.

**Phase 1: Data Input and Preprocessing.** The pipeline begins with the raw WM811k wafer maps. To ensure data consistency and enhance the model’s generalization capabilities, the input images undergo a rigorous preprocessing sequence. This includes resizing the images to a unified resolution of 224×224 pixels, applying data augmentation techniques (such as random flips, rotations, and color jittering) to simulate complex industrial conditions, and performing normalization.

**Phase 2: Proposed FCS-VMamba Architecture.** The preprocessed images are fed into the PatchEmbed2D module (with a stride of four) for tokenization. Subsequently, the features are processed through a four-stage hierarchical architecture. As depicted in the detailed FSSBlock in Figure 1, the core feature extraction relies on our three proposed mechanisms synergizing with the state space model. The Frequency Attention (FA) module precisely captures high-frequency structural energy anomalies via the RFFT amplitude; the SS2D core performs efficient 2D selective scanning; the Saliency Feature Suppression (SFS) module applies a detached soft mask to dynamically filter non-defect periodic textures; and the Cross-Layer Cross-Attention (CLCA) module salvages minute defect details from shallow layers. Patch merging is employed between stages to perform downsampling while preserving critical semantic information.

**Phase 3: Defect Classification Output.** In the final phase, the deep semantic features extracted by the Stage 4 FSSLayer are aggregated using global average pooling (GAP). A fully connected classification head then maps these aggregated features into a nine-dimensional space, accurately outputting the specific wafer defect categories (e.g., center, donut, or scratch).

### 3.2. VMamba Model

The VMamba architecture represents a novel visual backbone network based on the state space model (SSM), which is widely applied across diverse computer vision tasks including image classification, object detection, and semantic segmentation. This architecture centers on the Visual State Space (VSS) block to construct an efficient, scalable structure. Its core component, the 2D Selective Scan (SS2D) module, effectively bridges the gap between one-dimensional sequential scanning and two-dimensional spatial data processing. It captures global contextual information while maintaining linear time complexity. The VMamba architecture employs a multi-stage hierarchical feature extraction structure. This progressively reduces the image resolution while extracting multi-scale feature information. The SS2D module maintains efficient feature interaction, thereby capturing comprehensive spatial context and structural details.

Upon entering the VMamba architecture, images first pass through the stem module; a strided convolution divides the input image $R^{H\times W\times 3}$ into non-overlapping image blocks, generating a two-dimensional feature map with a spatial resolution H/4×W/4 without requiring additional positional embeddings. Subsequently, the feature map undergoes progressive feature extraction and resolution compression across four hierarchical stages. Each stage (except the first one) commences with a downsampling layer (implemented via strided convolution), halving the spatial resolution and adjusting the number of channels, followed by the stacking of multiple VSS blocks as the core feature learning units.

Each VSS block integrates an SS2D module, a deep convolutional layer (DWConv), and a feedforward network (FFN). The SS2D module traverses the feature map along four scan paths (Cross-Scan), converting two-dimensional spatial data into a sequence stream. Each stream is processed in parallel via selective scanning operations, followed by a merging operation (Cross-Merge) to capture global contextual information from multiple perspectives. DWConv enhances local spatial feature interactions, while the FFN enriches feature representations in the channel dimension.

After four processing stages, the feature map is compressed to a resolution of H/32×W/32, with the multi-scale features extracted at each stage fully utilized for downstream tasks. In image classification tasks, the final feature map undergoes global average pooling and a fully connected layer to output class probabilities. For dense prediction tasks such as object detection and semantic segmentation, hierarchical features are fed into task-specific head networks (e.g., Mask R-CNN for detection or UperNet for segmentation) for further feature fusion and prediction. Throughout this process, VMamba maintains linear computational complexity with respect to the input size, circumventing the quadratic complexity issue of self-attention in visual transformers. This achieves an excellent balance between inference speed and feature representation capability.

### 3.3. FCS-VMamba Model and Structural Enhancements

**Figure 2:**
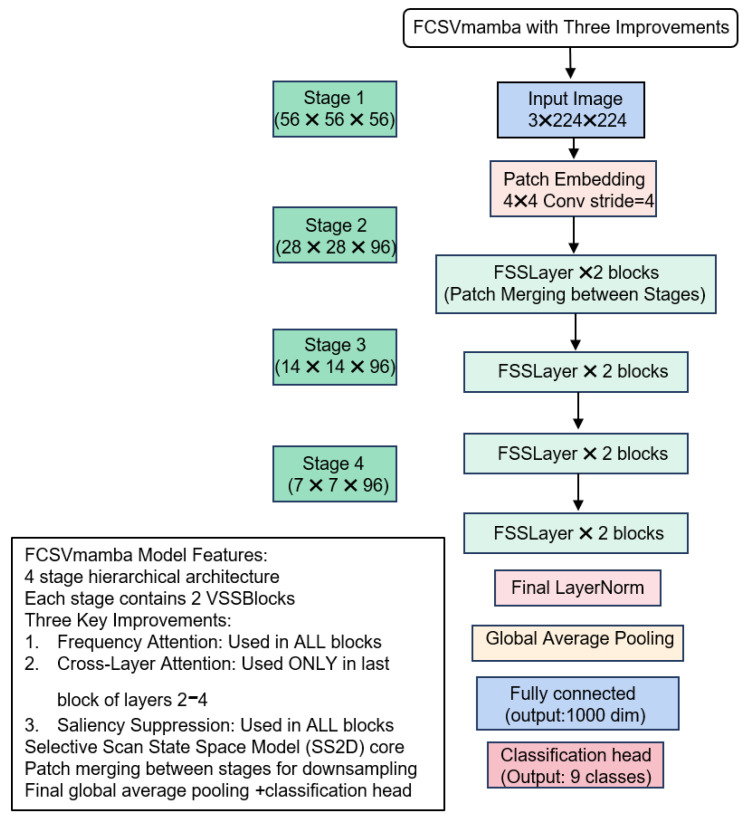
Architecture diagram of FCS-VMamba.

Building upon the efficient linear complexity foundation of the VMamba model, we introduce our customized architectural innovations. Figure 2 shows the FCS-VMamba network architecture that we independently constructed. The core components comprise Patch Embedding, an enhanced encoder (incorporating FSSLayer and Patch Merging2D), a Frequency Domain Attention module, a Salient Feature Suppression module, a Cross-Layer Cross-Attention module, a fully connected classification head, and residual connections.

**Figure 3:**
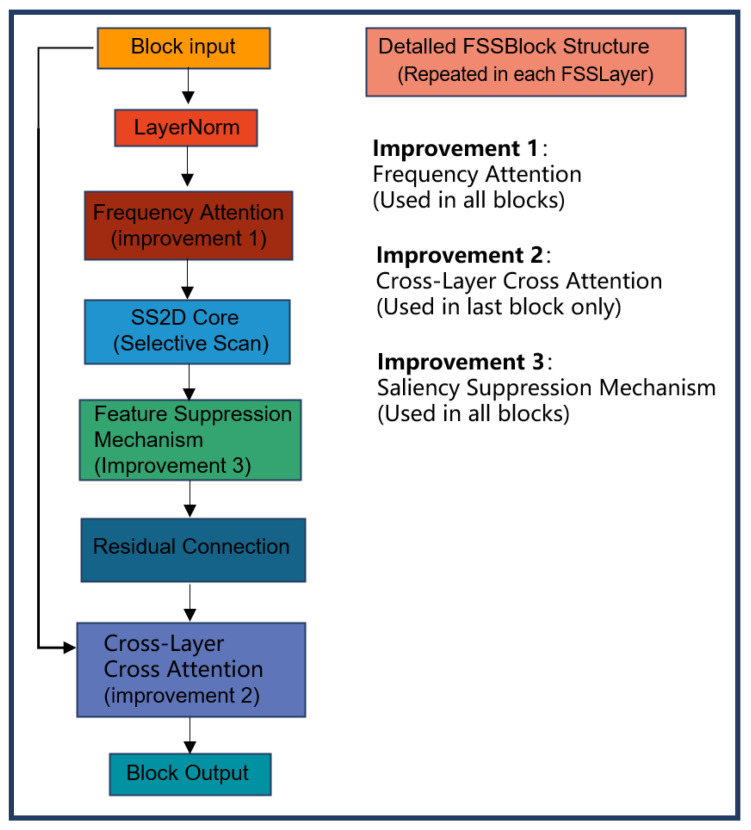
Structure diagram of the FSSLayer block.

First, Patch Embedding divides 224×224 high-resolution wafer images into 4×4 non-overlapping patches, embedding them into a 96-dimensional feature space to generate H/4×W/4×96 feature maps. This fully preserves the local details of defects such as nanoscale particles and shallow etching, laying the foundation for multi-scale feature extraction. Subsequently, the feature map undergoes multi-level progressive processing within an enhanced encoder. As shown in Figure 3, each encoder layer comprises multiple enhanced FSSLayers (derived from VMamba’s VSSLayer) and Patch Merging2D. The FSSLayer follows this workflow: LayerNorm feature normalization → FFT-based frequency domain attention modeling → SS2D linear complexity local feature capture → salient feature suppression module for micro-defect detection → residual connections preserving low-level information.

Specifically, the Frequency Attention (FA) module employs the 2D Real FFT (RFFT) amplitude spectrum to capture the global structure of periodic lithographic textures on wafers, resolving misclassification issues caused by texture interference and shallow etching non-uniformity.

The Salient Feature Suppression module attenuates highly activated prominent regions, compelling the model to focus on low-response minute defects. Cross-layer attention is embedded only in the final FSSLayer to efficiently fuse shallow-layer details with deep-layer semantic features.

Patch Merging2D implements layer-by-layer downsampling. To meet strict memory-efficient requirements for industrial edge deployment, we break convention by maintaining a fixed 96-dimensional channel count during subsampling (rather than the traditional doubling of channels), halving only the spatial resolution.

This reduces feature maps from H/4×W/4×96 to H/32×W/32×96, substantially enhancing the network’s dual capture capability of local defect details and global distribution patterns while controlling the parameter volume.

The high-dimensional features output by the encoder are integrated and fed into a fully connected classification head. This head maps features to the wafer defect category space, completing the transformation from feature representation to classification outcome. Residual connections traverse all encoder layers, directly adding the input to each FSSLayer’s processed features. This effectively mitigates the vanishing gradient problem in deep networks, ensuring the complete transmission of underlying defect features.

The superior performance of this entire architecture is primarily driven by the domain-specific theoretical reconstructions embedded within the network. In the following subsections, we detail these three core mechanisms, beginning with how we capture global structural anomalies.

### 3.4. Frequency Attention Module

Wafer surface defects often manifest as minute anomalies at the pixel or sub-pixel level, exhibiting highly sparse spatial distributions. FcaNet [44] theoretically demonstrated that conventional global average pooling (GAP) merely extracts the lowest-frequency direct current (DC) component and discards high-frequency structures. A mechanical transplantation of its discrete cosine transform (DCT) into our framework is fundamentally flawed for industrial inspection. The core of FcaNet relies on a multi-spectral 2D DCT for general image feature compression. However, wafer images possess unique physical characteristics; the background consists of highly periodic normal circuits, whereas defects are local anomalies with highly random spatial coordinates.

To strictly address this, we completely abandon the DCT approach and independently design a novel pooling mechanism based on the amplitude spectrum of the 2D real fast Fourier transform (2D RFFT). Unlike the DCT, which is sensitive to spatial translation, the amplitude spectrum of the FFT inherently possesses excellent mathematical shift-invariance. By applying GAP exclusively to the FFT amplitude spectrum, our FA module effectively extracts the “average frequency energy”. This crucial design allows the model to precisely capture the high-frequency structural energy anomalies triggered by nanoscale micro-defects, remaining completely undisturbed by their random spatial occurrences on the wafer. This represents a domain-specific mathematical reconstruction tailored to the wafer physics rather than a simple engineering transplant.

Furthermore, this customized FA module integrates this frequency-domain global information to guide spatial domain feature extraction within the VMamba backbone. While the state space model (SSM) block achieves efficient sequence feature extraction, its internal linear projection layer struggles to integrate global structural information. Our FA module overcomes this by converting frequency-domain energy anomalies into channel attention weights for globally guided feature enhancement.

The specific mathematical workflow is as follows. First, the FA module maps the spatial feature tensor’s $X\in R^{B\times C\times H\times W}$ output from the SSM block to the frequency domain via a 2D Real FFT, yielding the complex-valued spectral feature:

(1) $$F\left(X\right)={\mathrm{RFFT}}_{2D}\left(X\right)\in C^{B\times C\times H\times W^{\prime }}$$

where $W^{\prime }=\left\lfloor W/2\right\rfloor +1$ due to the conjugate symmetry of the real FFT.

After obtaining the spectral energy distribution via the modulus operation (i.e., calculating the amplitude spectrum to ensure shift invariance), spatial information is compressed using global average pooling (GAP) along the spatial dimensions to generate the frequency-aware channel descriptor (i.e., the average frequency energy) $g\in R^{B\times C\times 1\times 1}$, expressed as

(2) $$g_{c}=\frac{1}{H\times W^{\prime }}\sum_{i=1}^{H}\sum_{j=1}^{W^{\prime }}\left|F{\left(X\right)}_{b,c,i,j}\right|$$

Subsequently, the channel attention weights w are learned through a bottleneck structure comprising two linear transformations:

(3) $$w=\sigma \left(W_{2}\cdot \mathrm{ReLU}\left(W_{1}g\right)\right)$$

where $W_{1}\in R^{\frac{C}{r}\times C}$ denotes the dimension reduction matrix, $W_{2}\in R^{C\times \frac{C}{r}}$ represents the dimension expansion matrix, *r* is the compression ratio, and σ denotes the sigmoid activation function.

By utilizing the translation-invariant amplitude spectrum rather than relying on naive DCT components, our FA module comprehensively encodes structural information within the channel. Guided by this customized global frequency-domain energy, the FA module accurately focuses on micro-defect regions while aggressively suppressing the highly periodic texture interference of regular circuits.

### 3.5. Saliency Feature Suppression Mechanism

Following the frequency-domain modulation and local feature capture via the SS2D core, it is critical to explicitly address the dominant interference from the wafer’s background patterns to protect fragile defect signals.

The wafer surface itself exhibits highly repetitive circuit patterns and periodic textures, with certain regular structures potentially exhibiting greater “visual distinctiveness” statistically than genuine defects. This leads traditional models to over-rely on the most discriminative, high-response regions and misclassify non-defective textures as positive samples. Consequently, they readily overlook subtle, low-response defect features that are critical for accurate identification.

Generic saliency-guided suppression architectures (such as those used in person reidentification [45]) typically adopt a “hard erasure” strategy, which bluntly forces the feature values of high-response regions to zero. While effective for macroscopic objects containing a large number of pixels, directly applying hard erasure to the extremely sparse and minute micro-defects of a wafer instantly destroys the fragile feature distribution, inevitably leading to catastrophic gradient fragmentation and training collapse.

To fundamentally address this challenge, our theoretical innovation lies in the design of a “soft suppression with detached residuals” mechanism. Different from standard content-agnostic regularization methods (e.g., Dropout or Random Erasing) or conventional hard erasure, our SFS is a content-aware, deterministic regularizer engineered to insulate the gradient flow. The core principle is to emulate lateral inhibition effects in the primary visual cortex while mathematically guaranteeing a perfectly smooth loss surface.

The specific workflow is as follows. First, for the input feature map $X\in R^{B\times H\times W\times C}$, we compute the saliency distribution at each spatial location by aggregating the feature response intensity via a mean operation across the channel dimension:

(4) $$S=\frac{1}{C}\sum_{c=1}^{C}\left|X_{c}\right|\in R^{B\times H\times W\times 1}$$

Subsequently, we select the top k=αHW locations with the highest saliency (α being the suppression ratio) and construct suppression regions centered on these locations. To strictly avoid the gradient flow disruption caused by hard masking, our SFS innovatively implements a soft suppression strategy. We apply a mask M that explicitly retains 10% of the original feature intensity in highly activated areas (setting the mask value to 0.1 rather than 0), leaving other regions unchanged:

(5) $$M_{b,i,j}=\left\{\begin{array}{cc} 0.1 & \mathrm{if}\;\exists \left(m,n\right)\in T_{k}\;\mathrm{with}\;|i-m|\leq R\;\mathrm{and}\;|j-n|\leq R,\\ 1 & \mathrm{otherwise}. \end{array}\right.$$

where $T_{k}$ denotes the set of salient positions and *R* is the suppression radius. The mask M is multiplied element-wise with the original feature map to obtain the soft suppressed feature map $\dot{X}=X⊙M$.

Furthermore, to mathematically ensure a highly stable gradient flow during backpropagation, the final output Y is wrapped within a detached residual identity mapping:

(6) $$Y=X+(\dot{X}-X).\mathrm{detach}()$$

This proprietary design compels the network to bypass high-contrast periodic backgrounds during the forward pass to mine latent, widely distributed defect-related features. Experiments demonstrate that this mathematical reconstruction significantly enhances the model’s robustness against complex background interference.

### 3.6. Cross-Layer Cross-Attention

While the aforementioned SFS module effectively isolates minute defects from periodic backgrounds within a single layer, preventing these microscopic features from vanishing entirely across deep hierarchical stages necessitates a robust inter-layer interaction strategy.

Given the high resolution of wafer images and the wide defect size range (from a few pixels to tens of pixels), single-scale features struggle to balance detail preservation and semantic abstraction. While CrossViT [46] introduced cross-attention for feature interaction, a direct application of its architecture is theoretically misaligned with hierarchical models. CrossViT relies on a dual-branch parallel architecture (processing large and small patches simultaneously) to achieve parallel multi-scale fusion. However, state space models (such as VMamba) possess a critical architectural vulnerability: they perform highly aggressive spatial downsampling across hierarchical stages. In the context of semiconductor inspection, this rapid resolution reduction causes already minute, nanoscale defect features to completely vanish in deep network layers.

To fundamentally resolve this theoretical bottleneck, our CLCA approach entirely abandons the parallel branch design of CrossViT. Instead, we propose an innovative “serialized, vertical cross-layer interaction” mechanism. Rather than fusing parallel scales, our architecture executes a “vertical cross-layer salvaging” operation. As dictated by our underlying architectural logic, CLCA explicitly utilizes the high-resolution feature maps from the preceding hierarchical stage (the shallow layer) as the contextual key and value to directly respond to the query generated by the current stage (the deep layer). This fundamental, bottom-up redesign ensures that microscopic defect details, which are typically obliterated during downsampling, are forcibly salvaged and integrated into the deep, high-order semantics.

The bidirectional interaction is accomplished via a lightweight attention head with a linear computational complexity of O(N), avoiding the $O(N^{2})$ computational explosion associated with fully connected self-attention. First, the module receives the current layer’s features $x_{\mathrm{current}}\in R^{B\times H\times W\times C}$ and the previous layer’s features $x_{\mathrm{prev}}$. To establish exact spatial correspondence for the salvaging process, it spatially aligns $x_{\mathrm{prev}}$ via bilinear interpolation, yielding

(7) $$x_{\mathrm{aligned}}=\mathrm{Resize}\left(x_{\mathrm{prev}},\left(H,W\right)\right)$$

matching the current layer’s dimension.

Subsequently, the module constructs the attention mechanism through linear projections. Crucially, the query acts as the semantic anchor from the deep layer, while the key and value serve as the detailed spatial context salvaged from the shallow layer:

(8) $$Q=W_{q}\cdot \mathrm{Flatten}\left(x_{\mathrm{current}}\right),\;K=W_{k}\cdot \mathrm{Flatten}\left(x_{\mathrm{aligned}}\right),\;V=W_{v}\cdot \mathrm{Flatten}\left(x_{\mathrm{aligned}}\right)$$

Attention weights are computed following the scaled dot product rule. After normalization via the Softmax function, these weights are applied to the value for weighted fusion, achieving the targeted feature enhancement:

(9) $$\mathrm{Attention}\left(Q,K,V\right)=\mathrm{softmax}\left(\frac{QK^{T}}{\sqrt{d_{k}}}\right)V$$

During defect detection, deep layers identify overall structural deficiency trends, but their localization is inherently imprecise due to aggressive subsampling. By transitioning from CrossViT’s parallel multi-scale fusion to our serialized vertical cross-layer salvaging, deep semantic features dynamically retrieve exact spatial boundaries from shallow, high-resolution features. This explicitly mitigates the severe information decay within the SSM feature pyramid.

## 4. Experimental Set-Up and Result Analysis

### 4.1. Experimental Set-Up

All experiments were conducted on a server equipped with an NVIDIA RTX 4090 GPU (24 GB of VRAM) (NVIDIA Corporation, Santa Clara, CA, USA) running Ubuntu 22.04 LTS (Canonical Ltd., London, UK). The software environment was configured using PyTorch 2.1.0 and CUDA 12.1. The source code and datasets are publicly available at https://github.com/yijiazhang666/VMamba-for-semiconductor (accessed on 25 January 2026). For model optimization, the AdamW optimizer was employed with an initial learning rate of 0.001, a training batch size of 4, and a total of 50 training epochs.

### 4.2. Dataset Construction and Preprocessing

**Figure 4:**
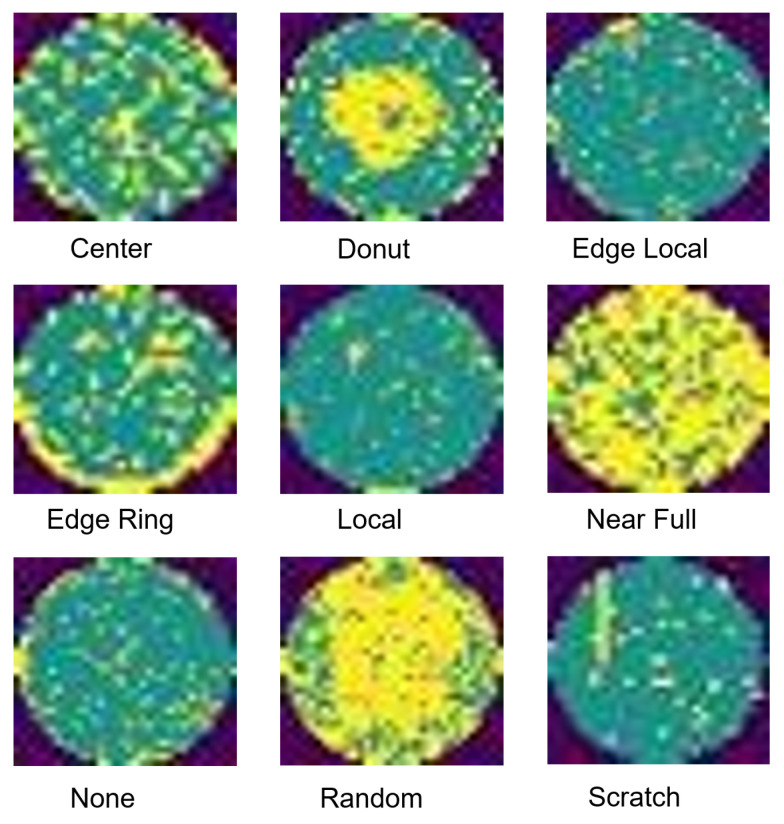
Nine types of wafer defects.

The original WM811k dataset contains over 810,000 wafer maps, but it suffers from extreme class imbalance (only approximately 172,950 are labeled) and inconsistent spatial dimensions. To rigorously evaluate the proposed FCS-VMamba architecture and address the inherent challenges of industrial deployment, we constructed a dual-level evaluation benchmark derived from the WM811k dataset:

(1) **Subset A: Balanced Benchmark (902 images).** To purely evaluate the feature representation capabilities of our proposed modules without the overwhelming interference of the majority class (defect-free wafers), we utilized a class-balanced subset. Following the random sampling methodology widely adopted in the field [47], we randomly sampled approximately 100 images for each of the nine categories (center, donut, edge local, edge ring, local, near full, none, random, and scratch), as illustrated in Figure 4. This subset prevents model bias toward majority classes and serves as the baseline for our ablation and comparative studies.
(2) **Subset B: Industrial Imbalanced Benchmark (11,000 images).** To validate the model’s robustness and scalability in true industrial environments, we randomly sampled a large-scale, highly imbalanced dataset without any manual intervention. This subset meticulously simulates the “high-yield, low-defect” reality of semiconductor fabrication lines, characterized by an extremely long-tailed distribution. Specifically, it comprised 11,000 images distributed as follows: none (8000), edge ring (1000), edge local (550), center (500), local (400), scratch (200), random (150), donut (100), and near full (100). The ratio of the largest class to the smallest class reached 80:1, posing a severe challenge for the model’s capacity to avoid majority class overfitting.

For both subsets, the training-to-validation ratio was strictly set to 8:2. To further enhance model generalization and prevent overfitting, data augmentation was applied during training, including random horizontal and vertical shifts, random rotations, and adjustments to the brightness, contrast, and saturation. It is important to note that Subset B (11,000 images) was utilized exclusively for the ablation experiments to evaluate industrial robustness, whereas all other evaluations, including the comparative studies and visualization analysis, were conducted on Subset A (902 images).

### 4.3. Evaluation Metrics

To ensure precise evaluation of the model performance, we employed multiple metrics to comprehensively measure the prediction accuracy: the confusion matrix, macro-precision, macro-recall, F1 score, and Top-1 recognition rate. Performance comparisons were conducted based on the FLOPs values, FPS, and model size.

#### 4.3.1. Confusion Matrix

**Figure 5:**
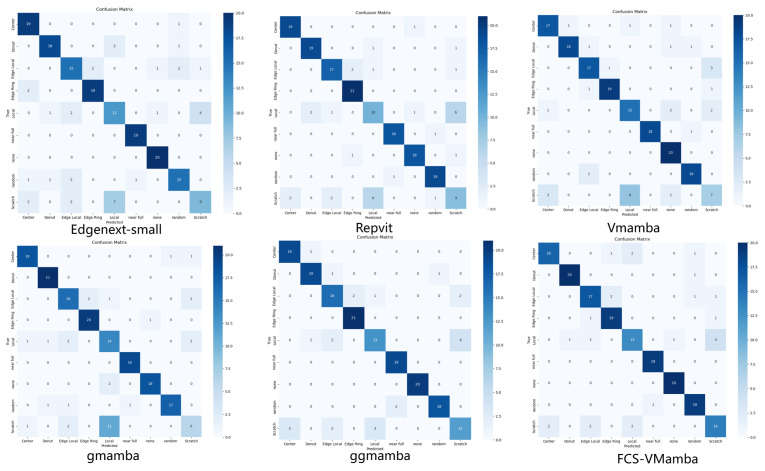
Confusion matrices of the six models.

Figure 5 (derived from Subset A) shows a two-dimensional table displaying the model’s predicted results for each category alongside their corresponding true labels. The confusion matrix provides an intuitive analysis of the model’s prediction bias across categories, and it served as the foundation for calculating metrics such as the precision and recall.

#### 4.3.2. Macro Precision

In multi-classification tasks, this metric first calculates the precision for each individual class and then takes the average of all class precisions. It reflects the model’s overall classification capability across all classes:

(10) $${\mathrm{Precision}}_{i}=\frac{{\mathrm{TP}}_{i}}{{\mathrm{TP}}_{i}+{\mathrm{FP}}_{i}}$$

(11) $$\mathrm{Macro-Precision}=\frac{1}{C}\sum_{i=1}^{C}{\mathrm{Precision}}_{i}$$

#### 4.3.3. Macro-Recall

In multi-classification tasks, the recall is first calculated for each category, and then the average of all categories’ recalls is taken. This measures the model’s average ability to identify positive samples across each category:

(12) $${\mathrm{Recall}}_{i}=\frac{{\mathrm{TP}}_{i}}{{\mathrm{TP}}_{i}+{\mathrm{FN}}_{i}}$$

(13) $$\mathrm{Macro-Recall}=\frac{1}{C}\sum_{i=1}^{C}{\mathrm{Recall}}_{i}$$

#### 4.3.4. F1 Score

The F1 score is the harmonic mean of the precision and recall. Its core function is to balance both metrics, avoiding the one-sidedness of relying on a single indicator:

(14) $${F1}_{i}=\frac{2\times {\mathrm{Precision}}_{i}\times {\mathrm{Recall}}_{i}}{{\mathrm{Precision}}_{i}+{\mathrm{Recall}}_{i}}$$

(15) $$\mathrm{Macro-}F1=\frac{1}{C}\sum_{i=1}^{C}{F1}_{i}$$

#### 4.3.5. Top-1 Accuracy

In classification tasks, this denotes the proportion of samples where the model’s highest-confidence prediction matches the true label. It serves as the most fundamental accuracy metric for classification tasks.

### 4.4. Ablation Studies

Ablation studies were conducted to investigate the impact of different components on model performance using the WM811k dataset, focusing on Frequency Attention, Cross-Layer Cross-Attention, and Saliency Feature Suppression. Table 1 (Subset A, 902 images) shows that introducing the Frequency Attention module alone improved the macro-precision by 0.29%. Further incorporation of Cross-Layer Cross-Attention yielded a 5.13% increase in macro-precision, substantially enhancing overall model performance. Finally, integrating the Saliency Feature Suppression mechanism resulted in a 5.39% improvement in macro-precision.

These experimental results demonstrate that the three modules—Frequency Attention, Cross-Layer Cross-Attention, and Saliency Feature Suppression—though introducing a slight increase in computational complexity (FLOPs), significantly reduced the number of parameters while delivering notable performance gains.

Furthermore, although the theoretical FLOPs of FCS-VMamba were slightly higher, this was a reasonable adjustment made based on the actual demands of industrial applications; in practical deployment, memory constraints have a far greater impact on application effectiveness than computational power. Through the collaborative effect of multiple modules, we successfully compressed the parameter size to an ultra-low 1.20 M, which effectively alleviated memory pressure in real-world work scenarios. Although sequence unfolding in the 2D Selective Scan module mathematically increased the theoretical FLOPs, the hardware-optimized parallel scanning mechanism ensured that this did not translate into high latency. Ultimately, FCS-VMamba achieved an empirical inference speed of 273.18 FPS, easily satisfying industrial real-time requirements.

As shown in Table 2, To rigorously verify the robustness of these modules under extreme industrial conditions, we further extended the ablation study to the highly imbalanced industrial benchmark (Subset B, 11,000 images). In this long-tail scenario, the baseline VMamba model exhibited performance degradation in minority classes, yielding a Macro-Recall of 77.49% and a Macro-F1 of 77.64%, reflecting the tendency of standard architectures to overfit the overwhelming majority class. The introduction of the Frequency Attention module increased the Macro-Precision to 80.78% and Macro-F1 to 81.62%, indicating that capturing high-frequency anomalies helps the model resist background noise. Notably, the further incorporation of Cross-Layer Cross-Attention delivered a substantial leap. This massive improvement mathematically proves that the vertical salvaging mechanism effectively preserves minority defect details that would otherwise be washed out by the dominant normal samples during aggressive downsampling. Ultimately, integrating the Saliency Feature Suppression module led to the best overall performance, peaking at a Macro-Precision of 85.89%, Macro-F1 of 86.23%, and Top-1 accuracy of 91.80%. By actively applying soft suppression to the heavily repeated normal circuit textures, the SFS module forced the network to maintain high sensitivity to rare defects.

### 4.5. Comparative Experiments

To ensure a fair and objective comparison of foundational feature representation capabilities among different mainstream architectures (CNNs, standard ViTs, and SSMs), comparative experiments were conducted on the balanced benchmark (Subset A, 902 images). This strategic choice isolated the intrinsic architectural effectiveness of each model from the compounding effects of extreme data skew, ensuring that the competitors were evaluated on a strictly level playing field. We compared our FCS-VMamba against two state-of-the-art parameter-efficient models: EdgeNeXt-Small and RepViT.

As shown in Table 3, the FCS-VMamba model achieved significant improvements across all comprehensive metrics. Specifically, the Macro-Precision reached 86.06%, surpassing the EdgeNeXt-Small model by 6.35 percentage points and the RepViT model by 4.48 percentage points. The Macro-Recall was 86.29%, exceeding EdgeNeXt-Small by 6.00 percentage points and RepViT by 3.94 percentage points. Consequently, the Macro-F1 score peaked at 86.06%, outperforming EdgeNeXt-Small and RepViT by 6.38 and 4.19 percentage points, respectively. The Top-1 accuracy also reached a leading 87.91%.

Although the theoretical FLOPs of our model were mathematically higher due to the sequential unfolding of the 2D Selective Scan (SS2D) module, it demonstrated highly competitive parameter efficiency (only 1.20M parameters). Being lightweight in industrial edge deployment is not solely dictated by FLOPs but also fundamentally constrained by the on-chip memory (SRAM) footprint. By keeping the parameters comparable to RepViT and lower than EdgeNeXt-Small, while delivering a highly practical empirical inference speed of 273.18 FPS, FCS-VMamba proved to be the most optimal and balanced architecture for high-precision wafer map classification.

### 4.6. Statistical Robustness and Cross-Validation

To strictly address the statistical standards and verify that the performance superiority of our proposed architecture was not due to random dataset splitting or lucky initialization, we conducted a robust statistical significance analysis. We adopted the Monte Carlo cross-validation (Repeated Random Sub-Sampling) strategy.

Specifically, we conducted five independent runs using different random seeds for dataset splitting and weight initialization. To isolate and evaluate the architectural improvements, this rigorous validation was focused on comparing the baseline VMamba and our final FCS-VMamba on the balanced benchmark. The statistical results, expressed as the mean ± standard deviation (SD), are summarized in Table 4.

As demonstrated in Table 4, the baseline VMamba exhibited noticeable performance fluctuations across different data splits, particularly suffering high variance in its Macro-Recall (SD = 2.28%). In stark contrast, FCS-VMamba not only elevated the absolute Macro-F1 performance by a massive margin of 5.27 percentage points but also maintained a strictly narrow standard deviation (1.65%). This statistically proves that the robustness and superiority of FCS-VMamba are deeply rooted in our structural innovations (Frequency Attention, Cross-Layer Cross-Attention, and SFS) rather than statistical coincidence.

### 4.7. Visualization Analysis

**Figure 6:**
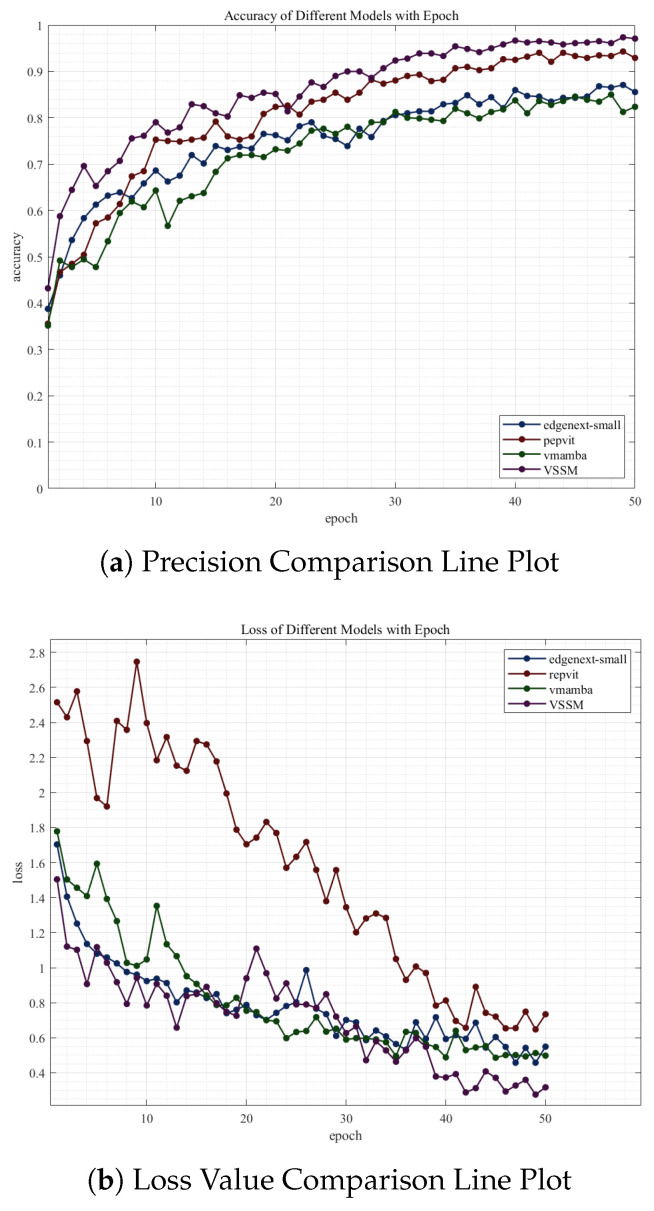
Comparison Line Plots: (**a**) precision comparison and (**b**) loss value comparison.

In Figure 6, to provide a more intuitive assessment of the improved model’s performance, we compare the training accuracy and training loss curves of the FCS-VMamba, EdgeNeXt-Small, RepViT, and VMamba models under identical experimental conditions.

The results demonstrate that the FCS-VMamba model exhibited a faster rate of accuracy increase during the initial training phase, with its loss function declining rapidly via steeper gradients and stabilizing earlier than its counterparts. The FCS-VMamba architecture achieved significantly faster accuracy growth than other models during the initial training, stabilizing above 95% accuracy as early as 40 iterations and maintaining a sustained lead thereafter. Although its loss rate was marginally lower than EdgeNeXt-Small and VMamba between 20 and 30 iterations, it declined substantially after 21 iterations and surpassed them after 32 iterations. This convergence behavior not only demonstrates the FCS-VMamba architecture’s high adaptability to wafer defect classification tasks but also further validates its comprehensive advantages in classification accuracy, optimization efficiency, and generalization stability. It better meets the practical demands for efficient and reliable classification models in semiconductor manufacturing scenarios.

## 5. Conclusions

In this paper, we proposed a novel, parameter-efficient FCS-VMamba classification network that addresses critical challenges in semiconductor wafer defect recognition, including inadequate modeling of long-range dependencies, false negatives for minute defects, complex background interference, and edge deployment constraints.

By innovatively integrating a Frequency Attention (FA) module, Cross-Layer Cross-Attention (CLCA), and Saliency Feature Suppression (SFS), the architecture achieved a synergistic optimization of its accuracy, footprint, and inference speed. Specifically, the FA module, based on the FFT amplitude spectrum, precisely captured the high-frequency characteristics of minute anomalies while maintaining spatial shift invariance. Furthermore, the CLCA mechanism facilitates bidirectional interaction between shallow-layer detail features and deep-layer semantic features through a serialized vertical salvaging mechanism, effectively mitigating information decay during aggressive downsampling.

The SFS mechanism acts as a content-aware regularizer, employing a soft-suppression strategy to dynamically filter non-defect interference, such as periodic wafer textures, without destabilizing the gradient flow. Its parameter-efficient design demonstrates strong potential for efficient inference in memory-constrained (SRAM) environments.

To rigorously validate the model, we evaluated it using a dual-level strategy. The experimental results demonstrate that on both the balanced subset and the highly imbalanced industrial benchmark of 11,000 images, FCS-VMamba achieved outstanding robustness. With only 1.2 million parameters, it achieved a Macro-Precision of 86.06% and Top-1 accuracy of 87.91%, successfully overcoming the tendency of standard models to overfit majority classes. This provides a practical, stable, and memory-efficient baseline for high-precision automated wafer map pattern classification in semiconductor manufacturing.

However, limitations remain. The model’s performance on physical edge hardware and its true generalization capability across different process nodes in real industrial scenarios require further empirical validation, and three-dimensional defect recognition issues remain unresolved.

Future research will focus on three key directions: (1) integrating multimodal sensor data to enhance sub-nanometer defect perception capabilities; (2) establishing process-adaptive mechanisms and conducting rigorous edge hardware testing for cross-environment zero-shot transfer learning; and (3) co-optimizing inspection-diagnosis workflows to provide interpretable root-cause analysis for wafer defects, ultimately advancing semiconductor manufacturing toward fully intelligent closed-loop control.

## Figures and Tables

**Table 1:** Ablation study on the balanced benchmark (Subset A, 902 images).

Model	Macro-Precision	Macro-Recall	Macro-F1	Top-1	FLOPs (G)	Params (M)	FPS
VMamba	81.66	81.88	81.27	83.52	10.27	46.02	674.36
gVMamba	81.90	81.83	81.21	85.16	10.27	46.81	594.75
ggVMamba	85.85	86.29	85.93	86.81	3.71	15.62	745.88
FCS-VMamba	86.06	86.29	86.06	87.91	13.71	1.20	273.18

Note: gVMamba added Frequency Attention only. ggVMamba added Frequency Attention and Cross-Layer Cross-Attention.

**Table 2:** Ablation study on the industrial imbalanced benchmark (Subset B, 11,000 images).

Model	Macro-Precision	Macro-Recall	Macro-F1	Top-1	FLOPs (G)	Params (M)	FPS
VMamba	79.66	77.49	77.64	86.50	10.27	46.02	674.36
gVMamba	80.78	83.01	81.62	87.20	10.27	46.81	594.75
ggVMamba	85.28	85.33	85.27	90.55	3.71	15.62	745.88
FCS-VMamba	85.89	87.40	86.23	91.80	13.71	1.20	273.18

**Table 3:** Comparison of results with state-of-the-art models on the balanced benchmark (Subset A, 902 images).

Model	Macro-Precision	Macro-Recall	Macro-F1	Top-1	FLOPs (G)	Params (M)	FPS
EdgeNeXt-Small	79.71	80.29	79.68	81.87	0.26	1.84	1216.69
RepViT	81.58	82.35	81.87	82.97	5.62	1.09	801.06
FCS-VMamba	86.06	86.29	86.06	87.91	13.71	1.20	273.18

**Table 4:** Statistical robustness evaluation across 5 independent runs (Monte Carlo Cross-Validation).

Model	Macro-Precision (Mean ± SD)	Macro-Recall (Mean ± SD)	Macro-F1 (Mean ± SD)
VMamba	81.49% ± 1.35%	80.83% ± 2.28%	80.64% ± 1.56%
FCS-VMamba	86.52% ± 1.60%	86.04% ± 1.09%	85.91% ± 1.65%

## Data Availability

The data presented in this study are openly available in GitHub at https://github.com/yijiazhang666/VMamba-for-semiconductor (accessed on 25 January 2026).
